# Chemical and Biological Analyses of the Essential Oils and Main Constituents of *Piper *Species 

**DOI:** 10.3390/molecules17021819

**Published:** 2012-02-13

**Authors:** Dominique F. Moura do Carmo, Ana Cláudia Fernandes Amaral, Gérzia M. C. Machado, Leonor Laura Leon, Jefferson Rocha de Andrade Silva

**Affiliations:** 1 Laboratório de Cromatografia, Departamento de Química, Universidade Federal do Amazonas, Av. Rodrigo Otávio, 3000, Coroado, CEP 69077-000, Manaus, AM, Brazil; Email: jrocha_01@ufam.edu.br (J.R.A.S.); 2 Laboratório de Plantas Medicinais e Derivados, Depto de Produtos Naturais, Farmanguinhos FIOCRUZ, Rua Sizenando Nabuco, 100, Manguinhos, CEP 21041-250, RJ, Brazil; Email: acamaral@fiocruz.br; 3 Laboratório de Bioquímica de Tripanosomatídeos, IOC, FIOCRUZ, Av. Brasil, 4365, Manguinhos, CEP 21045-900, RJ, Brazil

**Keywords:** *Piper duckei*, *Piper demeraranum*, essential oil, Leishmaniasis, HET-CAM

## Abstract

The essential oils obtained from leaves of *Piper duckei* and *Piper demeraranum *by hydrodistillation were analyzed by gas chromatography-mass spectrometry. The main constituents found in *P. demeraranum* oil were limonene (19.3%) and β-elemene (33.1%) and in *P. duckei* oil the major components found were germacrene D (14.7%) and *trans*-caryophyllene (27.1%). *P. demeraranum* and *P. duckei* oils exhibited biological activity, with IC_50_ values between 15 to 76 μg mL^−1^ against two *Leishmania* species, *P. duckei* oil being the most active. The cytotoxicity of the essential oils on mice peritoneal macrophage cells was insignificant, compared with the toxicity of pentamidine. The main mono- and sesquiterpene, limonene (IC_50_ = 278 μM) and caryophyllene (IC_50_ = 96 μM), were tested against the strains of *Leishmania amazonensis*, and the IC_50_ values of these compounds were lower than those found for the essential oils of the *Piper* species. The HET-CAM test was used to evaluate the irritation potential of these oils as topical products, showing that these oils can be used as auxiliary medication in cases of cutaneous leishmaniasis, with less side effects and lower costs.

## 1. Introduction

Leishmaniasis, one of the parasitic diseases spread worldwide, with about 12 million infected people, presents an increasing number of new cases [1]. In Brazil, studies report the occurrence of about 20.000 new cases of the illness annually [2]. Leishmaniasis is caused by parasites of the genus *Leishmania*, after transmission by phlebotominae (sandflies). The chemotherapeutic agents used for the treatment of leishmaniasis such as sodium stibogluconate, *N*-methylglucamine antimonate, pentamidine and amphotericin B are not orally active and require a long-term parenteral administration. These agents also present severe side effects such as cardio and renal toxicities and are expensive [3]. Additionally, parasites of the genus *Leishmania* are increasingly resistant to available antileishmanial agents, and so the identification of new compounds that could be active against these parasites is an urgent priority [4]. The majority of this research is based on the use of natural products.

The discovery of new drugs from natural products against various diseases, including parasitic diseases such as malaria and leishmaniasis [5,6,7] has gained new impetus in recent years, especially if we observe that a considerable amount of the drugs used today are derived from natural products [8,9]. In this respect, ethnopharmacology has an important role in the identification of medicinal plants with pharmacological potential [9,10]. Medicinal plants and their extracts have been used for many centuries to treat different diseases and their essential oils and/or active constituents can be a source of alternative or adjunct phytomedicines for the treatment of parasites. Many treatments, including topical therapy, have been suggested for this disease and in this respect, plant extracts may be effective [11].

The Hen’s Egg Test Chorioallantoic Membrane (HET-CAM) assay has been used to assess the irritation potential of substances [12] and plant extracts, and has proven useful in screening natural products [13]. These *in vivo* CAM screening tests were comparable to tests on human skin, and a similar level of efficacy was obtained [13].

*Piper* species are widely used in folk medicine in Latin America and the West Indies [14], to heal wounds, reduce swelling and skin irritations, and treat the symptoms of cutaneous leishmaniasis [15]. Several studies emphasize the importance of *Piper * species in the treatment of this disease [16,17,18,19,20,21]. In fact, various classes of antiparasitic active compounds have been identified in *Piper* species, such as chalcones and dihydrochalcones [22,23], benzoic acid derivatives [17] and neolignans [21].

The chemical composition of essential oils of *Piper* is mainly comprised of phenylpropanoids such as safrole, dillapiol and myristicin, or terpenes such as limonene, β-caryophyllene, spathulenol, (*E*)-nerolidol, α-bicyclogermacrene and cadinol [19,24,25]. Insecticidal, fungicidal, bactericidal, larvicidal and molluscicidal properties are attributed to these species [25]. Also, Guerrini and collaborators [19] carried out extensive pharmacological evaluation of essential oils obtained from *P. aduncum* and *P. obliquum*, with interesting results.

In this context, encouraged and inspired by reports that show the activity of *Piper* species against leishmaniasis, and by a few reports on *P. demeraranum* and *P. duckei* [26,27,28], we decided to carry out a study on these two species with frequent distribution in the Amazonas state of Brazilian Amazonia.

This present study investigated the chemical composition, antileishmanial and cytotoxic activities and irritation potential, through *in vivo* CAM assays, of essential oils obtained from leaves of *P. demeraranum* and *P. duckei*. Additionally, two main constituents, (−) limonene and caryophyllene, were tested against strains of *Leishmania amazonensis*.

### 2. Results and Discussion

#### Chemical Study

The yields of the essential oils obtained from the leaves of *P. demeraranum* and *P. duckei* were 0.6 and 0.5%, respectively. The optical rotations of the oils were *P. demeraranum* 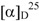 = −1.30° (CHCl_3_, *c* 1.75) and *P. duckei *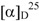 = +1.38° (CHCl_3_, *c* 1.30). Table 1 lists the constituents of the essential oils of *P. demeraranum* and *P. duckei* analyzed by GC-MS. Although aromatic compounds are common constituents in the essential oils produced by the Piperaceae species, none were detected in the oils analyzed. In total, 25 different compounds were identified and the results showed that the essential oils of these species are rich in sesquiterpenes.

**Table 1:** Chemical composition of the leaf essential oils of *P. demeraranum *and *P. duckei*.

Compounds		Oil Composition (%)	
	RI	*P. demeraranum*	*P. duckei*
**Monoterpenes**			
α−Pinene	932	3.9	0.7
β-Pinene	974	6.7	0.4
(-)-Limonene	1024	19.3	-
1,8 – Cineole	1026	-	5.8
**Sesquiterpenes**			
δ-Elemene	1335	1.0	0.1
α-Copaene	1374	0.5	1.6
β-Elemene	1389	33.1	-
*trans*-Caryophyllene	1417	6.0	27.1
β-Copaene	1430	-	0.5
α-Humulene	1452	-	2.5
( *Z*)-Muurola-4-(14),5-diene	1465	-	1.1
Germacrene-D	1484	*5.2*	14.7
β-Selinene	1489	5.0	-
Bicyclogermacrene	1497	8.8	5.2
α-Muurolene	1500	-	1.4
( *E,E*)-α-Farnesene	1505	1.0	-
γ-Cadinene	1513	-	1.8
δ-Cadinene	1522	1.3	3.9
α-Calacorene	1544	-	0.3
Germacrene B	1559	1.1	-
( *E*)-Nerolidol	1561	-	0.4
Guaiol	1600	-	1.1
1- *epi*-Cubenol	1627	-	0.6
γ-Eudesmol	1630	-	17.9
α-Muurolol	1644	-	3.0
Terpenoids classes			
Monoterpene hydrocarbons		29.9	1.1
Oxygenated monoterpenes		-	5.8
Sesquiterpene hydrocarbons		63.0	60.2
Oxygenated sesquiterpenes		-	23.0
**Identified Components (%)**		**92.9**	**90.1**

Regarding the content of monoterpenes when comparing the two species, *P. demeraranum* presented a higher concentration (29.9%), with limonene (19.3%) being its major compound. The main sesquiterpene of the essential oil of *P. demeraranum* was β-elemene (33.1%), while that of *P. duckei* was *trans*-caryophyllene (27.1%). Great amount of oxygenated sesquiterpenes was found only in the oil of *P. duckei *(23.0%). Santos *et al.* [26] collected *P. duckei* in another area of the State of Amazonas, and reported a different major constituent for essential oil and no monoterpenes, presumably due to the extraction process used. A total of 6.9% of monoterpenes were identified by us in the essential oil of *P*. *duckei*.

Also, 19 mono- and sesquiterpenes were detected, representing 29.9% and 63.0% of the total essential oil of *P*. *demeraranum*. This terpene class of *P. demeraranum *essential oil from the State of Amazonas was therefore different when compared with the species collected in the State of Pará by Andrade *et al.* [28]. In addition, there are other factors that influence the difference between these results, which are not part of this study. Here, the sesquiterpene β-elemene (33.1%) was the major constituent, followed by the monoterpene limonene (19.3%), the major constituent reported by Andrade *et al.* [28] for Pará oil.

Both *Piper* essential oils inhibited the growth of promastigote forms in the two species of *Leishmania* tested, with IC_50_ of between 15 and 76 μg mL^−1^. The essential oils of *P. duckei* (IC_50_ = 15.2 μg mL^−1^) and *P. demeraranum* (IC_50_ = 22.7 μg mL^−1^) indicated greater activity against *L*. *guyanensis * promastigotes and weakened inhibition of *L*. *amazonenis* (Table 2). These oils inhibited the growth of amastigote forms of *L. amazonensis*, again showing more activity than the *P. duckei* oil (IC_50_ = 42.4 μg mL^−1^)*. *The non-parasitized peritoneal macrophages were preserved at the concentrations of the essential oils, which were toxic to the parasite. Even when used at concentrations seven times higher, both essential oils were less toxic than pentamidine, proving to be highly relevant for future use.

Various studies have demonstrated significant activity of mono- and sesquiterpenes against *Leishmania* species [5,29], but few studies have been conducted using essential oil from *Piper* species [20]. The antileishmanial activity of *P. demeraranum *oil may be associated with the main monoterpene limonene, which has been linked to several biological activities [30,31], including antimalarial [32] and antileishmanial activity [33]. Some sesquiterpenoids have been described as promising substances for the treatment of leishmaniasis. In fact, several oxygenated sesquiterpenes have evidenced significant activity against *Leishmania *species [5,7]. These reports are consistent with the activity found for these substances in this work.

**Table 2:** Leishmanicidal and Cytotoxicity Activities of *Piper demeraranum *and *P. duckei *Essential Oils.

Samples	*L. amazonensis*		*L. guyanensis*	Mice (Balb/c) macrophages	
	*Promastigotes*	*Amastigotes*	*Promastigotes*		
	IC_50_ ± SD	IC_50_ ± SD (µg/mL)	IC_50_ ± SD (µg/mL)	Cytotoxicity / (%)	
*P. demeraranum*	86.0 ± 2.4 µg/mL	78 ± 1.0	22.7 ± 1.1	IC_50_ 7 × IC_50_	14.422.3
*P. duckei*	46.0 ± 1.3 µg/mL	42.4 ± 0.8	15.2 ± 0.9	IC_50_ 7 × IC_50_	16.824.4
Limonene	278 ± 12 µM	-	-	-	-
*trans* -Caryophyllene	96 ± 19 µM	-	-	-	-
Pentamidine	4.6 ± 0.4 µg/mL	-	0.8 ± 0.2	-	26.2

The main mono- and sesquiterpene of the oils, (−) limonene and *trans*-caryophyllene, respectively, were evaluated against promastigote forms of *L. amazonensis* and showed IC_50_ value of 278 μM and 96 μM, respectively, both being more active than their corresponding essential oils. Among these activities, the antileishmanial activity of *trans*-caryophyllene was at least two times more potent than limonene. The inhibitory effect of limonene against *L. amazonensis *was similar to that described by Arruda and collaborators [33], but this is the first description of caryophyllene with this *Leishmania* species.

It is worth mentioning that during the experiments, it was observed that the purity of caryophyllene is an important factor for the activity against *L*. *amazonensis*. The oxidation of caryophyllene into its corresponding oxides affects the results and depending on this oxidation level, the activity cannot be observed (data not shown).

At this point, it was found that the oils under study were not toxic; that they showed antileishmanial activity; and that they can be obtained from renewable parts of the plant. Therefore, based on the popular use of *Piper* species for the treatment of skin wounds, further assays were conducted to evaluate the potential of these oils for use as topical medicine. The HET-CAM assay was used to show the irritation potential of these essential oils because it is rapid and inexpensive. The oils were applied without complications. The data from this assay were quite promising, featuring oils with slight and moderate skin irritations at a dose calculated to be about 10 times the IC_50_ of *L*. *amazonensis*. The results of the irritation induced by essential oils are described in Table 3. Water was used as the negative reference substance and a second control, 0.1% DMSO + water (used to dissolve the oils), was used and did not provoke any reaction. A positive control such as sodium dodecyl sulphate (SDS) 1% was also tested. The concentration used for both oils did not cause bleeding or clotting of the membrane. The essential oils of *P. duckei* and *P. demeraranum* had an irritation score (IS) of 4.9 and 5.1, indicating a slightly irritant and moderately irritant effect, respectively, as described by Luepke [12] at concentrations above those observed for parasite growth inhibition.

**Table 3:** Assessment of irritation potential of *Piper oils *tested in the CAM.

Samples	Congestion	Haemorrhage	Coagulation	Irritation score	Irritation Assessment
Control (-)	0	0	0	0	Non-irritant
2^sd^ Control	0	0	0	0	Non-irritant
Control (+)	5	5.7	4.7	15.4	Strong
PDU (500 µg)	1.8	0	0	4.9	Slight
PDE (900 µg)	1.7	0	0	5.1	Moderate

Control (+): sodium lauryl sulfate 1%; Control (−): water; 2^sd^ Control: water + DMSO 0.1%; PDU: *P. duckei*; PDE: *P. demeraranum*.

These results are promising for *in vivo* tests, especially if one considers the low density and high lipophilicity of these oils and their components, which have rapid transdermal penetration, facilitating their action [34]. Another important point is the presence of a terminal methylene group in the structure of the substances tested, described as an important site that has harmful effects on the microorganisms [35]. Nevertheless, further studies are required to explain the complete mechanisms of these interactions, particularly in the specific case of leishmaniasis.

The discovery of topical medications for the treatment of leishmaniasis has increased, gaining support from various agencies, such the World Health Organization (WHO). These results are relevant, since the development of new phytotherapeutic agents containing essential oils, or their active compounds, as well as their application in cutaneous leishmaniasis, can have a positive impact on this worldwide neglected disease, with less side effects and lower costs.

## 3. Experimental

### 3.1. Plant Material

The specimens of *P. duckei* (PDU) and *P. demeraranum* (PDE) were collected in the Adolpho Ducke reserve, Km 26 Manaus – Itacoatiara highway, in the State of Amazonas, Brazil. Voucher specimens were deposited at the Herbarium of the Institute of Biological Sciences (UFAM) under the numbers 188224 (PDU) and 188187 (PDE).

### 3.2. Extraction of Essential Oil

The leaves of *P. duckei* and *P. demeraranum *(600 g) were dried at room temperature, ground, and submitted to hydrodistillation (4 h) using a modified Clevenger-type apparatus. After the end of each distillation, the oils were collected, transferred to glass flasks, and stored at a temperature of −4 °C. The yields were calculated based on the weight of the plant material used. (−) Limonene (96%) and *trans*-caryophyllene (98.5%) were supplied by Sigma –Aldrich Chemical Co and Fluka, respectively.

### 3.3. Essential Oil Analysis

The oil composition was performed by comparison of their retention indices and mass spectra with those reported in the literature [36] or stored in the Wiley data system library. The retention indices were calculated for all volatile constituents using *n*-alkane homologous series. GC-MS analyses were performed using an Agilent 6890N gas chromatograph interfaced with an Agilent 5973N Mass Selective Detector (ionization voltage 70 eV), fitted with a DB-5MS column (30 m × 0.25 mm, film thickness 0.25 μm), using helium as the carrier gas at 1.0 mL min^−1^. The oven temperature was programmed from 60 °C to 290 °C at a rate of 3 °C min^−1^, and hydrogen was used as carrier gas (1.0 mL min^−1^). Injector and detector temperatures were 230 °C and 280 °C, respectively. Optical rotations were measured in 15 mm cell at 25° C at 589 nm (sodium D-Line) with a digital polarimeter JASCO DIP-370.

### 3.4. Anti-leishmanial Assay

Promastigotes of *Leishmania amazonensis* (MHOM/BR/77LTB0016 strain) and *L. guyanensis *(MHOM/BR/95/IOCL-2092/IM4216 strain) isolated from patients with cutaneous leishmaniasis, the latter in Manaus, were routinely cultured at 26ºC in Schneider’s medium [37] supplemented with 10% fetal calf serum (FCS), pH 7.2.

Parasites (promastigotes) were harvested from the medium on day three of culture, resuspended in fresh medium, counted in a Neubauer chamber, and adjusted to a concentration of 4 × 10^6^ parasites mL^−1^, for the drug assay. The essential oils to be tested were dissolved in DMSO (the highest concentration was 1.4%, which was not hazardous to the parasites) and added to parasite suspensions at a final concentration of between 0.156 and 320 μg mL^−1^. The standards of limonene and caryophyllene were dissolved in DMSO at concentrations of 60, 30, 15 and 7.5 μg/mL. After 24 h of incubation, the parasites were counted and compared with the controls containing DMSO and parasites alone. All the tests were done in triplicate and Pentamidine isethionate was used as the reference drug. The concentration causing 50% inhibition (IC_50_) was obtained from the drug concentration—response curve and the results were expressed as the mean ± standard deviation determined from three independent experiments [38,39].

Approximately 4 × 10^6^ BALB/c mice peritoneal macrophages were placed in a 24-well plate in complete RPMI medium (Sigma Cell Culture, St. Louis, MO, USA). *L*. *amazonensis* promastigote forms in a stationary phase of culture growth were added to the macrophage at a ratio of 10 parasites to 1 macrophage. The culture was incubated at 5% CO_2_ at 35 °C and after 24 h, the plate was washed with phosphate buffer solution (PBS) at 25 °C to remove non-internalized promastigotes. A new culture medium at 25 °C was added and the essential oil was added to the infected macrophages at concentrations of 60, 30, 15 and 7.5 μg mL^−1^. After 24 h, the infected macrophages were fixed and stained with Giemsa to verify the amastigote counts within the macrophages [40].

### 3.5. Cytotoxicity Assay

In order to evaluate the toxicity of the sample for the host cell, mice peritoneal macrophages were isolated in RPMI 1640 medium, containing 200 UI mL^−1^ penicillin, 200 μg mL^−1^ of streptomycin, 1 mM sodium piruvate, 1 mM of L-glutamine and 1M HEPES buffer (Sigma Cell Culture, St. Louis, MO, USA). Cells were counted in Neubauer’s chamber using erythrosine B as vital dye (Sigma Cell Culture) and adjusted to a concentration of 4 × 10^6^ cells mL^−1^. Cells were then cultured in a 96 wells culture plate (Falcon, NJ, USA) at 37 °C and in an atmosphere of 5% CO_2_. The sample was added to the medium in a concentration equivalent to IC_50_ and 7× IC_50_ of the *in vitro* activity assay from *L. guyanensis*. The sample and pentamidine isethionate (reference drug) were added to the culture and after 24 h, the viability of treated cells was compared to that of the control without drugs, through the MTT methodology [41].

### 3.6. Statistical Analysis

Statistical significance (*p* < 0.05) was evaluated by the ANOVA test followed by Student-Newman-Keuls, by Kruskall-Wallis or Mann-Whitney tests software SPSS for Windows.

### 3.7. HET-CAM Test *(*Hen’s Egg Test—Chorioallantoic Membrane*)*

The method of Luepke [12] was used. The test samples were dissolved and dropped onto the chorioallantoic membrane at a volume of 0.2 mL, followed by the controls. In every case, a series of five eggs was used. After the application of the sample, the CAM, the blood vessels, including the capillary system, and the albumen were examined. The ensuing irritation reaction was observed over 5 min, with the time (sec) to first appearance of each hemorrhage, lysis and coagulation recorded as in irritancy assays (congestion, haemorrhage, coagulation). According to Lupke [12], the results can be expressed in the following categories: non-irritant (0.0–0.9), slight irritant (1.0–4.9), moderate irritant (5.0–8.9) and strong irritant (9.0–21.0).

## 4. Conclusions

In general, the acquisition of essential oils from plants is an uncomplicated and low cost procedure. Among the oils studied in this article, there is an obvious difference in the chemical constituents of them. The essential oil of *Piper duckei* as well as its main component, *trans*-caryophyllene, were the most active against *Leishmania* species. Further significant points, an insignificant toxicity and a low level of irritability observed for the oils analyzed, confirming possible utilization of these oils or compounds as auxiliary medication in cases of cutaneous leishmaniasis.
